# Organic Broadband THz Generators Optimized for Efficient Near‐Infrared Optical Pumping

**DOI:** 10.1002/advs.202001738

**Published:** 2020-09-03

**Authors:** Myeong‐Hoon Shin, Won Tae Kim, Se‐In Kim, Seung‐Jun Kim, In Cheol Yu, Sang‐Wook Kim, Mojca Jazbinsek, Woojin Yoon, Hoseop Yun, Fabian Rotermund, O‐Pil Kwon

**Affiliations:** ^1^ Department of Molecular Science and Technology Ajou University Suwon 443‐749 Korea; ^2^ Department of Physics Korea Advanced Institute of Science and Technology (KAIST) Daejeon 34141 Korea; ^3^ Institute of Computational Physics Zurich University of Applied Sciences (ZHAW) Winterthur 8401 Switzerland; ^4^ Department of Chemistry and Department of Energy Systems Research Ajou University Suwon 443‐749 Korea

**Keywords:** nonlinear optics, organic crystals, terahertz waves

## Abstract

New organic THz generators are designed herein by molecular engineering of the refractive index, phonon mode, and spatial asymmetry. These benzothiazolium crystals simultaneously satisfy the crucial requirements for efficient THz wave generation, including having nonlinear optical chromophores with parallel alignment that provide large optical nonlinearity; good phase matching for enhancing the THz generation efficiency in the near‐infrared region; strong intermolecular interactions that provide restraining THz self‐absorption; high solubility that promotes good crystal growth ability; and a plate‐like crystal morphology with excellent optical quality. Consequently, the as‐grown benzothiazolium crystals exhibit excellent characteristics for THz wave generation, particularly at near‐infrared pump wavelengths around 1100 nm, which is very promising given the availability of femtosecond laser sources at this wavelength, where current conventional THz generators deliver relatively low optical‐to‐THz conversion efficiencies. Compared to a 1.0‐mm‐thick ZnTe crystal as an inorganic benchmark, the 0.28‐mm‐thick benzothiazolium crystal yields a 19 times higher peak‐to‐peak THz electric field with a broader spectral bandwidth (>6.5 THz) when pumped at 1140 nm. The present work provides a valuable approach toward realizing organic crystals that can be pumped by near‐infrared sources for efficient THz wave generation.

## Graphical abstract

Organic crystals, which are designed through molecular engineering of the refractive index, phonon mode, and spatial asymmetry, simultaneously satisfy the crucial requirements for efficient and broadband THz wave generation, particularly at near‐infrared pump wavelengths near 1100 nm.

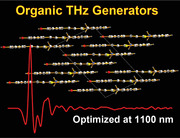

## 1 Introduction

Efficient generation of broadband THz waves is essential for diverse applications in the THz frequency range. Many recent studies have confirmed that THz waves generated in nonlinear optical organic crystals by optical rectification or difference frequency generation processes provide excellent THz generation characteristics.^[^ ^1^, ^2^, ^3^, ^4^, ^5^, ^6^, ^7^, ^8^ ^]^ When pumped in the infrared region of 1300‒1500 nm, the optical‐to‐THz conversion efficiency of the state‐of‐the‐art nonlinear optical organic crystals is more than one order of magnitude higher than that of widespread conventional nonlinear inorganic crystals such as ZnTe and GaP, with a substantially broader spectral bandwidth.^[^ ^1^, ^2^, ^3^, ^9^, ^10^ ^]^ Furthermore, compared to the tilted‐pulse‐front technique with LiNBO_3_ that is often used for high‐power THz systems, organic THz generators afford much simpler setup in a single‐pass configuration at room temperature and deliver broader THz spectra.^[^ ^1^, ^11^, ^12^ ^]^

However, developing efficient broadband organic THz generators for near‐infrared pumping at wavelengths close to the 1 µm region is rather difficult. This is because nonlinear organic crystals must simultaneously satisfy many requirements for optimized frequency conversion. To develop organic THz generators, one of the most crucial undertakings is the suitable design of new nonlinear optical organic crystals with large macroscopic optical nonlinearity. This can be achieved by the parallel alignment of highly polar nonlinear optical chromophores in the crystalline state. However, the strong tendency toward centrosymmetric dipole−dipole aggregation of nonlinear optical chromophores in many cases results in loss of the macroscopic optical nonlinearity. Only a few types of nonlinear organic crystals with large macroscopic nonlinear optical susceptibilities (e.g., effective hyperpolarizability $\beta_{ijk}^{\mathrm{eff}}$ > 90 × 10^−30^ esu) have been reported.^[^ ^3^, ^6^, ^13^, ^14^ ^]^

To obtain high optical‐to‐THz conversion efficiency and a broad spectral bandwidth, good phase‐matching between the optical pump and THz wavelengths generated by the organic crystals should be achieved.^[^ ^2^ ^]^ Most currently available benchmark nonlinear organic crystals provide high optical‐to‐THz conversion efficiency under optical pumping in the infrared region of 1300‒1500 nm;^[^ ^1^, ^2^, ^3^, ^9^, ^10^ ^]^ however, at near‐infrared wavelengths of around (and below) 1100 nm, they exhibit inferior phase matching compared to that achieved at longer wavelengths.^[^ ^15^, ^16^ ^]^ Note that the ability to pump around 1100 nm is very promising owing to the technological importance of this wavelength, where diverse high‐power femtosecond laser sources at or around this wavelength have been developed, including the recent development of compact high‐power lasers such as Yb:fiber lasers and thin‐disk lasers.^[^ ^16^, ^17^ ^]^

In addition to the large macroscopic optical nonlinearity and good phase matching at near‐infrared wavelengths close to the 1 µm region, nonlinear organic crystals should also possibly satisfy additional requirements. For instance, inducing strong intermolecular (and interionic) interactions is an important step in suppressing the phonon and vibrational modes (i.e., restraining THz self‐absorption).^[^ ^18^ ^]^ Furthermore, a plate‐like crystal morphology with good optical quality based on good crystal growth ability is desired for optical experiments.

In this work, new nonlinear optical organic crystals are designed through molecular engineering of the refractive index, phonon mode, and spatial asymmetry to achieve efficient broadband THz wave generation. The as‐grown benzothiazolium crystals can simultaneously satisfy the above‐mentioned requirements. When pumped at near‐infrared wavelengths of 1140 nm, the peak‐to‐peak THz electric field of the new organic crystals is up to 19 times higher than that of standard ZnTe, with a substantially broader bandwidth with an upper cut‐off THz frequency of >6.5 THz.

## 2 Results and Discussion

### 2.1 Design of Molecular Salt Crystals

**Figure 1:**
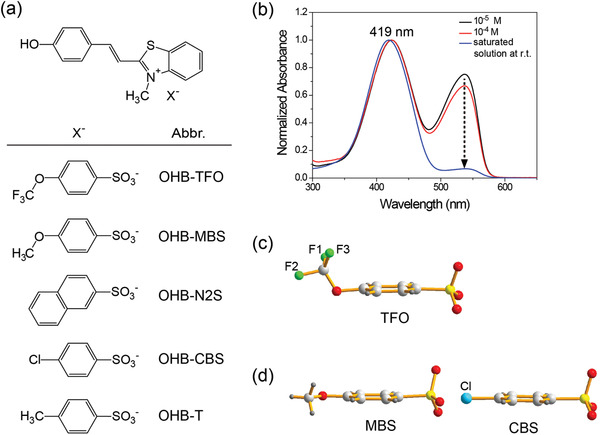
a) Chemical structure of OHB‐based crystals. b) UV‐VIS absorption spectra of OHB‐TFO in methanol at different concentrations. Molecular conformation of c) nonplanar TFO anion in OHB‐TFO crystals and d) planar MBS and CBS anions in OHB‐MBS and OHB‐CBS crystals, respectively.

**Figure** **1**a shows the chemical structure of the investigated nonlinear optical organic crystals. The crystals consist of the cationic chromophore 2‐(4‐hydroxystyryl)‐3‐methylbenzothiazol‐3‐ium (OHB) and one of the five selected benzenesulfonate counter anions. As mentioned above, state‐of‐the‐art nonlinear optical organic crystals show poor phase matching between the near‐infrared optical pump (typically ≤ 1140 nm) and the generated THz waves. One potential approach for improving the phase‐matching characteristics of such crystals for near‐infrared optical pumping is to introduce a wide bandgap chromophore, accompanied with refractive index engineering, which can lead to a reduced optical group index in this wavelength range.^[^ ^15^ ^]^ The benzothiazolium‐based OHB chromophore exhibits a relatively wide bandgap between the highest occupied molecular orbital (HOMO) and the lowest unoccupied molecular orbital (LUMO) compared to benchmark nonlinear optical organic crystals consisting of low bandgap chromophores. As shown in Figure 1b, the wavelength of maximum absorption (*λ*_max_ = 419 nm) of the benzothiazolium‐based OHB chromophores with a hydroxy electron donor in methanol is much shorter than that of benchmark crystals: 438 nm for benzothiazolium‐based HMB (2‐(4‐hydroxy‐3‐methoxystyryl)‐3‐methylbenzothiazol‐3‐ium) with a hydroxy methoxy electron donor, 523 nm for benzothiazolium‐based PMB (2‐(4‐(4‐(hydroxymethyl)piperidin‐1‐yl)styryl)‐3‐methylbenzothiazol‐3‐ium) with a piperidino electron donor, and 439 nm for quinolinium‐based HMQ (2‐(4‐hydroxy‐3‐methoxystyryl)‐1‐methylquinolinium) crystals with hydroxy methoxy electron donor.^[^ ^6^ ^]^ Note that the absorption band at 419 nm corresponds to the phenolic benzenoid form, while the absorption band at 537 nm is related to the phenolate quinoid form. The phenolate absorption band at 537 nm only appears when this compound is in low concentration in solution. Therefore, in the crystalline state, the absorption band at 419 nm is expected to dominate the spectrum of the OHB‐based crystals.

As previously reported,^[^ ^15^ ^]^ good phase matching between the near‐infrared optical pump and the generated THz waves can be achieved for benzoimidazolium‐based HMI (2‐(4‐hydroxy‐3‐methoxystyryl)‐1,3‐dimethyl‐1H‐benzoimidazol‐3‐ium) crystals designed through refractive index engineering with a wide bandgap chromophore. However, HMI‐based crystals showed low photostability in prior THz generation experiments.

To obtain noncentrosymmetric alignment of the cationic chromophores and consequently macroscopic second‐order optical nonlinearity, five benzenesulfonate counter anions were introduced, i.e., TFO (4‐(trifluoromethoxy)benzenesulfonate), MBS (4‐(methoxy)benzenesulfonate), N2S (naphthalene‐2‐sulfonate), CBS (4‐chlorobenzenesulfonate), and T (4‐methylbenzenesulfonate) anions, leading to the corresponding OHB‐TFO, OHB‐MBS, OHB‐N2S, OHB‐CBS, and OHB‐T crystals, respectively. Introducing planar counter anions (MBS, N2S, CBS, and T) is a molecular design tool that is widely used to obtain noncentrosymmetric alignment of cationic chromophores in crystals.^[^ ^3^, ^19^ ^]^ However, this molecular salt approach does not fulfill all the requirements of efficient broadband THz generators. For instance, in OHB‐T crystals that were recently reported by Shi et al.,^[^ ^20^ ^]^ although OHB‐T crystals exhibited a noncentrosymmetric crystal symmetry group *Pca2*_1_, the OHB chromophores exhibited close to antiparallel alignment, which led to very small macroscopic second‐order optical nonlinearity.

In contrast to the planar four counter anions (MBS, N2S, CBS, and T), the TFO anion possesses unique features. In TFO anions, trifluoromethyl group that are highly electronegative, can undergo strong interionic binding interactions that suppress the phonon vibrational modes.^[^ ^18^, ^21^ ^]^ This so‐called phonon mode engineering leads to restrained THz self‐absorption.^[^ ^18^ ^]^ Furthermore, one conformation of the trifluoromethoxy group is nonplanar (i.e., spatial asymmetry), originating from an orthogonal orientation relative to the phenyl ring.^[^ ^21^ ^]^ In previous reports, asymmetric molecules exhibit a higher probability of noncentrosymmetric molecular alignment in crystals.^[^ ^13^, ^22^ ^]^ The spatial asymmetric shape of the TFO anions may also lead to increased solubility, with a large free volume, which is strongly related to the crystal growth ability.

### 2.2 Parallel Alignment of Chromophores

The orientation of the chromophores in the different OHB crystals was investigated by powder second‐harmonic generation (SHG) measurements and single crystal structure analysis. In the quantitative powder SHG analysis^[^ ^23^, ^24^ ^]^ at the fundamental wavelength of 1500‒1800 nm, which generates nonresonant SHG signals, the OHB‐TFO crystalline powder exhibited a strong SHG signal, whereas the OHB‐CBS and OHB‐T crystalline powders exhibited relatively weak SHG signals (see Figure S1, Supporting Information). The SHG signal generated by the OHB‐TFO crystalline powder was one order of magnitude stronger than that achieved with the OHB‐CBS and OHB‐T crystalline powders. From the qualitative powder SHG experiments at 1300 nm, the OHB‐N2S and OHB‐MBS crystals were found to be polymorphic. The OHB‐N2S powder that was recrystallized from methanol exhibited an SHG signal, whereas the OHB‐N2S crystals grown by the rapid cooling method in methanol did not show any indication of SHG. For OHB‐MBS (the recrystallized powder and crystals grown by the rapid cooling method), most of the samples did not show SHG signals, and only a minor part of the samples showed an SHG signal. Thus, among the five investigated crystals, the OHB‐TFO crystals were considered the most promising for nonlinear optical applications, including THz wave generation.

To investigate the molecular ordering of the OHB‐based crystals, the crystal structures of the OHB‐TFO, OHB‐MBS, and OHB‐CBS single crystals were analyzed (see details in Supporting Information). The OHB‐TFO and OHB‐CBS crystals crystallized with noncentrosymmetric symmetry in the *Cc* and *Pca2*_1_ space groups, whereas the OHB‐MBS crystals belong to the centrosymmetric space group *C2/c*. Note that the crystal structure of the OHB‐CBS crystals is very similar to that of previously reported OHB‐T crystals.^[^ ^20^ ^]^

Figure 1c,d displays the molecular conformation of the anions in the OHB‐TFO, OHB‐MBS, and OHB‐CBS crystals. Interestingly, although the TFO and MBS anions are analogues with an identical number of atoms, their conformations are remarkably different. The TFO anion is nonplanar, with orthogonal orientation of the trifluoromethoxy group (Figure 1c), whereas the MBS anion is planar, similar to the CBS anion (Figure 1d). The reason of different conformation between nonplanar TFO anion and planar MBS anion is related to the conformation of nonplanar trifluoromethoxybenzene and planar methoxybenzene.^[^ ^21^, ^25^ ^]^

**Figure 2:**
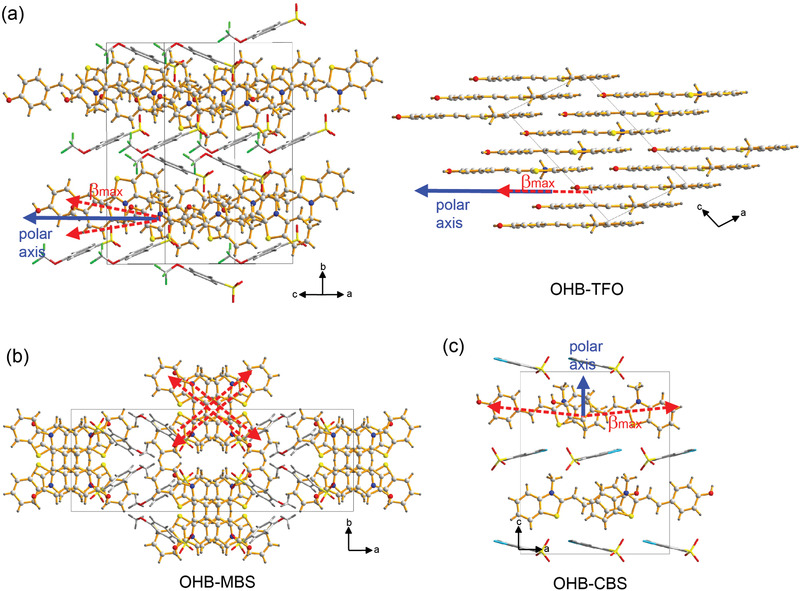
Molecular alignment of a) noncentrosymmetric OHB‐TFO, b) centrosymmetric OHB‐MBS, and c) noncentrosymmetric OHB‐CBS crystals. The red arrows represent the direction of the maximal first hyperpolarizability (*β*_max_) of the OHB cationic chromophores.

**Figure** **2** shows the molecular alignment of the OHB‐TFO, OHB‐MBS, and OHB‐CBS crystals. In the OHB‐TFO crystals with the nonplanar TFO anion, the OHB chromophores are aligned in an almost parallel orientation, with a small molecular ordering angle of *θ*_p_ = 10°, where the molecular ordering angle is defined as the angle between the polar axis of the crystal and the direction of the maximal first hyperpolarizability (*β*_max_) of the OHB cationic chromophores (indicated by red arrows in Figure 2), which is assumed to be directed from the O atom on the electron donor to the C atom between the S and N atoms on the electron acceptor. In contrast, in the OHB‐MBS and OHB‐CBS crystals with the planar MBS and CBS anions, the OHB chromophores adopt an antiparallel (or close to antiparallel) alignment, with a very large molecular ordering angle (*θ*_p_ = 90° for OHB‐MBS and ≈84° for OHB‐CBS). Note that OHB‐MBS crystals exhibit a centrosymmetric crystal structure, therefore OHB‐MBS crystals cannot be used as THz generators because of the absent second‐order optical nonlinearity. As discussed above, in acentric OHB‐TFO crystals, the hydrogen bond ability of —CF_3_ group may be beneficial for suppressing phonon vibrational modes (restrained THz self‐absorption).^[^ ^18^, ^21^ ^]^

The macroscopic optical nonlinearity was evaluated by estimating the maximal first hyperpolarizability (*β*_max_) and the effective hyperpolarizability tensors ($\beta_{ijk}^{\mathrm{eff}}$) for the OHB‐TFO crystals from the SHG intensity relative to that of the well‐known DAST (4‐(4‐(dimethylamino)styryl)‐1‐methylpyridinium 4‐methylbenzenesulfonate) crystal^[^ ^26^ ^]^ via quantitative powder SHG measurements at 1500‒1800 nm (Figure S1, Supporting Information). For this evaluation, we considered the spatially averaged effective hyperpolarizability components for the powder SHG efficiency in a 1D chromophore approximation^[^ ^3^, ^23^, ^27^ ^]^ and the hyperpolarizability components of the DAST chromophore, as determined by quantum chemical calculations.^[^ ^28^ ^]^ The details are described in Section D (Supporting Information). The resulting maximal first hyperpolarizability (*β*_max_) of the OHB cation in the OHB‐TFO crystals was 100 ± 7 × 10^−30^ esu. The diagonal component of the effective hyperpolarizability tensor of the OHB‐TFO crystals was large with a value of $\beta_{333}^{\mathrm{eff}}$= 96 × 10^−30^ esu, whereas the largest off‐diagonal component of the effective hyperpolarizability tensor was $\beta_{223}^{\mathrm{eff}}$= 3 × 10^−30^ esu. The diagonal optical nonlinearity of the OHB‐TFO crystals ($\beta_{333}^{\mathrm{eff}}$= 96 × 10^−30^ esu) is higher than that of the wide‐bandgap HMI‐based crystals ($\beta_{111}^{\mathrm{eff}}$= 77 × 10^−30^ esu) that show good phase‐matching characteristics for THz wave generation with near‐infrared pumping.^[^ ^15^ ^]^

### 2.3 Excellent Crystal Characteristics for THz Generation

**Figure 3:**
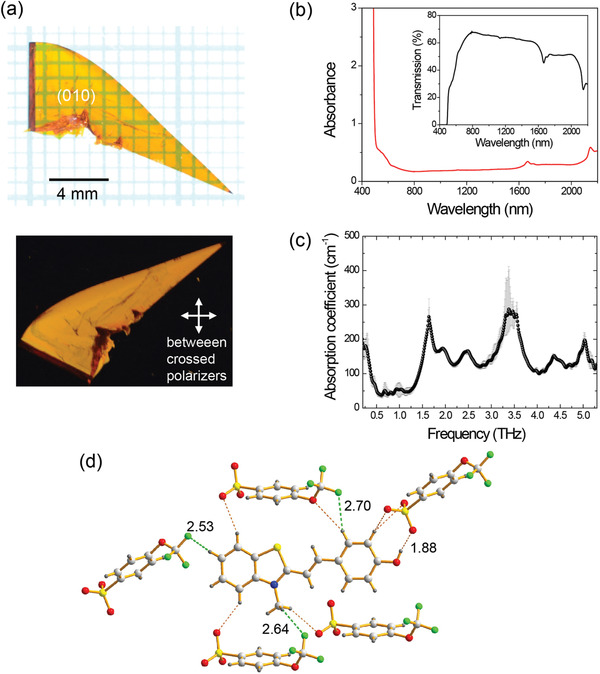
a) Photograph of as‐grown OHB‐TFO crystals. b) Absorbance (and transmittance in the inset) of a 0.39‐mm‐thick OHB‐TFO crystal, measured using unpolarized light. c) Absorption coefficient of OHB‐TFO crystals in the THz region. d) Interionic hydrogen bonds (—CF_3_⋅⋅⋅H—, —OH⋅⋅⋅H—, and —SO_3_^−^⋅⋅⋅H—) between TFO anions and OHB cations in OHB‐TFO crystals, with a distance of less than 3.0 Å.

In addition to the large macroscopic optical nonlinearity with a wide bandgap, the OHB‐TFO crystals exhibited excellent crystal characteristics for efficient THz wave generation. OHB‐TFO shows relatively high solubility: 10.48 g/100 g methanol and 1.87 g/100 g ethanol at 40 °C, where the solubility is much higher than that of most benchmark nonlinear optical organic crystals.^[^ ^6^, ^19^, ^29^ ^]^ This is attributed to the large free volume of the nonplanar TFO anions with orthogonal orientation of the trifluoromethoxy group (see Figures 1c and 2a). The high solubility of OHB‐TFO results in good crystal growth. **Figure** **3**a shows photographs of an OHB‐TFO crystal grown by the slow cooling method in ethanol.

The as‐grown OHB‐TFO crystals exhibit a plate‐like morphology that is suitable for most optical experiments, including THz wave generation, and also possess large lateral dimensions, allowing for a large aperture size. Note that the plate‐like morphology of the OHB‐TFO single crystals is very promising because the plate‐like crystals do not require any additional polishing and cutting processes or special crystal growth methods (e.g., confined geometry method^[^ ^30^ ^]^). To grow plate‐like single crystals, the confined geometry method is often useful as a second best option for organic crystals that naturally grow in a nonplate morphology or with nonoptimal thickness for THz wave generation.^[^ ^30^ ^]^ In a previous study of TFO‐containing analogous crystals, a confined geometry method was needed to grow single crystals with a plate‐like morphology with a large aperture.^[^ ^21^ ^]^ In addition, the facet of the as‐grown OHB‐TFO was excellent and the largest surface was the symmetry (010) plane (see Figure 3a). Therefore, the polar axis is in the plane of the largest surface of the as‐grown OHB‐TFO crystals, which allows the maximum projection of the pump beam polarization in the THz generation setup. Note that previously reported as‐grown benzothiazolium‐based PMB crystals exhibit a plate‐like morphology. However, the polar axis of PMB crystals is not parallel to the largest surface, and hence, the PMB crystals must be rotated for better access to the polar axis.^[^ ^6c^ ^]^

The crystal morphology of as‐grown OHB‐TFO single crystals may still be improved. Compared to the triangular crystal shape in Figure 3a, the square shape of single crystals is more optimal for obtaining larger aperture size in optical experiments.

Figure 3b shows the absorbance and transmission curves of an as‐grown 0.39‐mm‐thick OHB‐TFO single crystal. The OHB‐TFO single crystals exhibit optical transparency over a wide range without substantial scattering, indicating good optical quality. As photographically shown in Figure 3a, the uniform transmission of the as‐grown OHB‐TFO single crystal also confirmed the good optical quality.

The solubility of organic materials generally increases with increasing free volume (and flexibility of the substituents) in the crystalline state. However, increasing the free volume may result in stronger molecular vibrations, which leads to THz self‐absorption, and is therefore not desired. Despite the high solubility of the OHB‐TFO single crystals, the strong interionic binding interactions in these crystals may suppress these molecular phonon and vibrational modes.^[^ ^18^, ^21^ ^]^ Figure 3c shows the absorption coefficient of the OHB‐TFO crystals in the THz frequency range. Despite the high solubility of the OHB‐TFO crystals, their absorption coefficients are comparable to those of benchmark nonlinear optical organic crystals.^[^ ^9^ ^]^ This is mainly attributed to the strong interionic binding interactions in the former. For example, Figure 3d shows the interionic hydrogen bonds (—CF_3_⋅⋅⋅H—, —OH⋅⋅⋅H—, and —SO_3_^−^⋅⋅⋅H—) between the TFO anions and OHB cations in the OHB‐TFO crystals, with a distance of less than 3.0 Å. Thus, the OHB‐TFO single crystals with large macroscopic optical nonlinearity, wide bandgap, and plate‐like crystal morphology and resulting good optical quality are considered as a new potential candidate for efficient THz wave generation.

### 2.4 Efficient Broadband THz Generators

**Figure 4:**
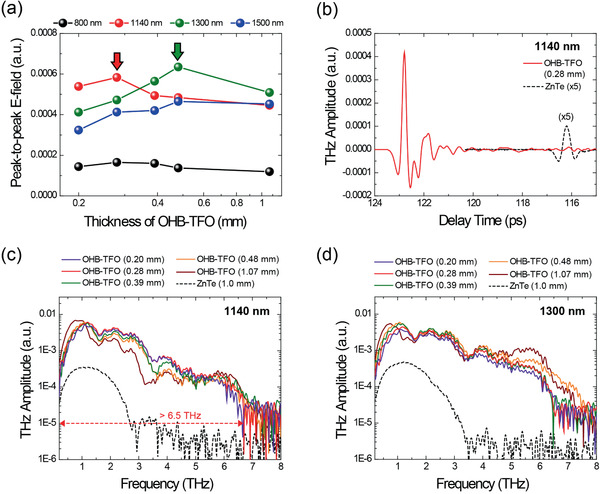
THz wave generation in as‐grown OHB‐TFO crystals with various thicknesses and in 1.0‐mm‐thick ZnTe crystals. a) Peak‐to‐peak THz electric field as a function of crystal thickness. b) Time traces obtained with 1140‐nm pumping. c,d) THz frequency spectra with pumping at 1140 nm (c) and 1300 nm (d).

The as‐grown OHB‐TFO crystals with various crystal thicknesses were directly used for THz wave generation. The THz waves were generated by optical rectification at various optical pump wavelengths (800, 1140, 1300, and 1500 nm) with a beam size of ≈1 mm and an average pump power of 20 mW. The optical pump pulses for optical rectification with a pulse duration of 150 fs above 1100 nm were generated from an optical parametric amplifier (TOPAS Prime‐F, Spectra Physics) pumped by a 1‐kHz Ti:sapphire regenerative amplifier (Spitfire Ace, Spectra Physics), while pulses with a pulse duration of ≈100 fs at 800 nm were directly generated from this amplifier. The generated THz waves were subsequently detected by an electrooptic sampling (EOS) technique similar to that used elsewhere.^[^ ^12^ ^]^ The pump beam and detected THz beam polarizations were along the polar axis of the as‐grown crystals in all cases. The results of the THz wave generation experiments are shown in **Figure** **4**.

Figure 4a shows the generated peak‐to‐peak THz electric field for the OHB‐TFO crystals as a function of the crystal thickness. The highest optical‐to‐THz frequency conversion efficiency of OHB‐TFO crystal was achieved at the pump wavelength of 1300 nm (see green arrow with 0.48‐mm thickness in Figure 4a), similar with benchmark nonlinear optical organic crystals.^[^ ^3^, ^9^ ^]^ Interestingly, at the near‐infrared pump wavelength of 1140 nm, the OHB‐TFO crystal also exhibited very high conversion efficiency, comparable to that at 1300 nm (see red arrow with 0.28‐mm thickness in Figure 4a). To evaluate the obtained efficiency, the widely used inorganic benchmark ZnTe crystal was used as a reference for comparison. Note that to confirm the conversion efficiency qualitatively, a widespread and well‐accepted method is the direct comparison of the THz waves generated in two different crystals under a same experimental condition.^[^ ^9^ ^]^ Compared to the 1.0‐mm‐thick inorganic ZnTe crystal, the peak‐to‐peak THz electric field of the 0.28‐mm‐thick OHB‐TFO crystal at the near‐infrared pump wavelength of 1140 nm was 19 times higher (Figure 4b) and the spectral bandwidth of the latter was 2‒3 times broader, with an upper cut‐off THz frequency of >6.5 THz (Figure 4c). This high optical‐to‐THz conversion efficiency under near‐infrared pumping around 1100 nm is technologically meaningful because diverse high‐power femtosecond laser sources of this wavelength are commercially available or are being developed.^[^ ^16^, ^17^ ^]^

Note that THz waves were also efficiently generated by OHB‐TFO crystal with 800 nm pumping. The peak‐to‐peak THz electric field of the 0.28‐mm‐thick OHB‐TFO crystal at the near‐infrared pump wavelength of 800 nm was ≈3 times higher than that generated with the 1.0‐mm‐thick inorganic ZnTe crystal.

The optical‐to‐THz conversion efficiency of the OHB‐TFO crystals is comparable to (or higher than) that of benchmark nonlinear optical organic crystals having larger optical nonlinearity than the OHB‐TFO crystals. The above estimated diagonal effective hyperpolarizability tensor coefficient of OHB‐TFO crystals ($\beta_{333}^{\mathrm{eff}}$= 96 × 10^−30^ esu), which is the component used for THz wave generation in this work, is large. However, it is still smaller than that of benchmark nonlinear optical organic crystals:, e.g., 185 × 10^−30^ esu for HMQ‐TMS (2‐(4‐hydroxy‐3‐methoxystyryl)‐1‐methylquinolinium 2,4,6‐trimethylbenzenesulfonate) and 274 × 10^−30^ esu for PMB‐T (2‐(4‐(4‐(hydroxymethyl)piperidin‐1‐yl)styryl)‐3‐methylbenzothiazol‐3‐ium 4‐methylbenzenesulfonate) crystals.^[^ ^6^, ^31^ ^]^ Nevertheless, when pumped at 800 nm, 0.33‐mm‐thick PMB‐T and 0.16‐mm‐thick HMQ‐TMS crystals have been reported comparable (or smaller) optical‐to‐THz conversion efficiency than 0.28‐mm‐thick OHB‐TFO crystal.^[^ ^6^, ^31^ ^]^ From the THz wave generation at near‐infrared optical pump wavelengths (800 and 1140 nm), we conclude that the OHB‐TFO crystals afford good phase matching under near‐infrared pumping.

Further, no photostability issues were observed under the present experimental conditions at a pump power of 20 mW with a 1.0‐mm aperture at 800 nm (corresponding fluence: 2.54 mJ cm^−2^). This is a much higher pump fluence than the damage threshold previously reported for benzoimidazolium‐based HMI crystals designed through refractive index engineering (0.8 mW with a 1.5‐mm aperture at 800 nm, leading to a fluence level of 0.045 mJ cm^−2^).^[^ ^15^ ^]^

The OHB‐TFO crystals also exhibited thickness‐independent THz generation characteristics. For the OHB‐TFO crystals, the maximum optical‐to‐THz conversion efficiency was achieved with the 0.28‐mm‐thick crystals when pumped in the near‐infrared (800 and 1140 nm) and with the 0.48‐mm‐thick crystals when pumped at longer wavelengths of 1300 and 1500 nm, as shown in Figure 4a. However, the thickness‐dependence of the optical‐to‐THz conversion efficiency for the OHB‐TFO crystals observed in this experiment was relatively small (Figure 4a), and the generated THz spectra also varied only slightly (Figure 4c,d). In many organic nonlinear optical crystals, THz wave generation characteristics exhibit large variation with the crystal thickness.^[^ ^3^, ^31^ ^]^ The thickness‐independent THz wave generation characteristics of the OHB‐TFO crystals may afford great freedom for crystal growth approaches. As mentioned above, for growing plate‐like morphology of the OHB‐TFO single crystals, there is no need for special crystal growth methods (e.g., confined geometry method^[^ ^30^ ^]^). During crystal growth for many benchmark organic nonlinear optical crystals, the thickness of the crystal (related to the beam path length) increases as the size of the crystal (related to aperture) increases. In many cases, the as‐grown crystals are large, but with nonoptimal thicknesses for THz wave generation. Therefore, the thickness‐independent THz generation characteristics of the OHB ‐TFO crystals are very beneficial.

## 3 Conclusion

OHB‐TFO crystals, designed through simultaneous engineering of the refractive index, phonon mode, and spatial asymmetry, exhibit efficient broadband THz wave generation, particularly at near‐infrared wavelengths around 1100 nm, as well as infrared wavelengths beyond 1100 nm. The engineered OHB‐TFO crystals simultaneously satisfy the primary requirements for THz wave generation, including possessing large optical nonlinearity with good phase matching between the optical pump and THz waves, strong interionic binding interactions, high solubility, excellent crystal growth ability, plate‐like crystal morphology, and good optical quality. Therefore, the OHB‐TFO crystals are considered a highly desirable new candidate for efficient and broadband THz wave generation.

## 4 Experimental Section

See the Supporting Information for details of the synthesis, powder SHG measurements, crystal structure analysis, and macroscopic optical nonlinearity.

## Conflict of Interest

The authors declare no conflict of interest.

## Supporting information

Supporting Information
