# Supplementary material for: Organic Broadband THz Generators Optimized for Efficient Near‐Infrared Optical Pumping
Source: Adv Sci (Weinh). 2020 Sep 3;7(20):2001738. doi: 10.1002/advs.202001738 (PMC7578856; doi:10.1002/advs.202001738)
Supplement: Supplementary file 1 — Supporting Information [file ADVS-7-2001738-s001.pdf]

© 2020 Wiley-VCH GmbH

ADVANCED SCIENCE
Open Access

Supporting Information

for *Adv. Sci.*, DOI: 10.1002/advs. 202001738

Organic Broadband THz Generators Optimized for Efficient Near-Infrared Optical Pumping

Myeong-Hoon Shin, Won Tae Kim, Se-In Kim, Seung-Jun Kim, In Cheol Yu, Sang-Wook Kim, Mojca Jazbinsek, Woojin Yoon, Hoseop Yun, Fabian Rotermund, and O-Pil Kwon**

Supporting Information

Organic Broadband THz Generators Optimized for Efficient Near-infrared Optical Pumping

Myeong-Hoon Shin,[†] *Won Tae Kim*[†]*, Se-In Kim, Seung-Jun Kim, In Cheol Yu, Sang-Wook Kim, Mojca Jazbinsek, Woojin Yoon, Hoseop Yun, Fabian Rotermund*, O-Pil Kwon**

((Optional Dedication))

M. H. Shin, S. I. Kim, S. J. Kim, Prof. S. Kim, Prof. O. P. Kwon
Department of Molecular Science and Technology, Ajou University, Suwon 443-749 (Korea)
E-mail: opilkwon@ajou.ac.kr
W. T. Kim, I. C. Yu, Prof. F. Rotermund
Department of Physics, Korea Advanced Institute of Science and Technology (KAIST), Daejeon 34141 (Korea)
E-mail: rotermund@kaist.ac.kr
Dr. M. Jazbinsek
Institute of Computational Physics, Zurich University of Applied Sciences (ZHAW), 8401 Winterthur (Switzerland)
W. Yoon, Prof. H. Yun
Department of Chemistry & Department of Energy Systems Research, Ajou University, Suwon 443-749 (Korea)

[†]These authors contributed equally to this work.

A. Synthesis

OHB-TFO, OHB-MBS, OHB-CBS, and OHB-T were synthesized via the condensation reaction, which is more suitable for synthesizing larger amounts compared to metathesis reaction. This is because for the synthesis of the final product, the condensation reaction uses small molecules (and intermediates) that are often highly soluble in organic solvents. In contrast, the metathesis reaction is often used bigger molecules (i.e., chromophores) with a relatively low solubility. The cationic chromophore OHB is synthesized via the condensation reaction between 4-hydroxybenzaldehyde, that consists of the electron donor phenol group, and 2,3-dimethylbenzothiazol-3-ium, that consists of the electron acceptor benzothiazolium group. All reactions are performed in methanol in the presence of a piperidine catalyst, as described below.

2-(4-Hydroxystyryl)-3-methylbenzothiazol-3-ium 4-(trifluoromethoxy)benzenesulfonate (OHB-TFO): 2,3-Dimethylbenzothiazol-3-ium 4-(trifluoromethoxy)benzenesulfonate (2.50 g, 6.2 mmol) and 4-hydroxybenzaldehyde (0.75 g, 6.2 mmol) were dissolved in methanol (15 mL) and piperidine (0.12 mL, 1.2 mmol). The solution was stirred at 50 °C for 27 h. The precipitated powder was filtered and purified by recrystallization in ethanol. An orange powder was obtained. Yield = 54%. ^{1}H NMR (600 MHz, DMSO-d_6, δ): 10.57 (s, 1H, C_6H_5O), 8.34 (d, 1H, J = 7.8 Hz, C_6H_4), 8.16 (d, 1H, J = 8.4 Hz, C_6H_4), 8.10 (d, 1H, J = 15.6 Hz, C_2H_2), 7.91 (d, 2H, J = 9 Hz, $C_6H_4F_3SO_4^-$), 7.81 (t, 1H, J = 7.8 Hz, C_6H_4), 7.77 (d, 1H, J = 15 Hz, C_2H_2), 7.72 (t, 1H, J = 7.8 Hz, C_6H_4), 7.66 (d, 2H, J = 8.4 Hz, $C_6H_4F_3SO_4^-$), 7.26 (d, 2H, J = 8.4 Hz, C_6H_5O), 6.90 (d, 2H, J = 9 Hz, C_6H_5O), 4.27 (s, 3H, $C_4H_3SN^+$). ^{13}C NMR (DMSO-d_6, δ): 718.68, 162.69, 149.86, 148.64, 148.27, 142.53, 133.08, 129.72, 128.62, 128.16, 127.95, 125.94, 124.61, 120.73, 117.09, 116.85, 110.62. Elemental analysis of $C_{23}H_{18}F_3NO_5S_2$: calcd. C 54.22, H 3.56, N 2.75, S 12.59; found: C 54.29, H 3.58, N 2.76, S 12.62.

2-(4-Hydroxystyryl)-3-methylbenzothiazol-3-ium 4-methoxybenzenesulfonate (OHB-MBS): 2,3-Dimethylbenzothiazol-3-ium 4-methoxybenzenesulfonate (1.00 g, 2.8 mmol) and 4-hydroxybenzaldehyde (0.35 g, 2.8 mmol) were dissolved in methanol (7 mL) and piperidine (0.03

mL, 0.3 mmol). The solution was stirred at 50 °C for 6 h. The precipitated powder was filtered and purified by recrystallization in methanol. An orange powder was obtained. Yield = 26%. ^{1}H NMR (400 MHz, DMSO-d_6): 10.63 (s, 1H, C_6H_5O), 8.38 (d, 1H, J = 7.6 Hz, C_6H_4), 8.19 (d, 1H, J = 8.4 Hz, C_6H_4), 8.13 (d, 1H, J = 15.6 Hz, C_2H_2), 7.95 (d, 2H, J = 8.8 Hz, $C_7H_7SO_4^-$), 7.84 (t, 1H, J = 7.8 Hz, C_6H_4), 7.81 (d, 1H, J = 16 Hz, C_2H_2), 7.75 (t, 1H, J = 7.7 Hz, C_6H_4), 7.51 (d, 2H, J = 8.8 Hz, $C_7H_7SO_4^-$), 6.94 (d, 2H, J = 8.8 Hz, C_6H_5O), 6.84 (d, 2H, J = 8.8 Hz, C_6H_5O), 4.30 (s, 3H, $C_4H_3SN^+$), 3.74 (s, 3H, $C_7H_7SO_4^-$). ^{13}C NMR (DMSO-d_6, δ): 172.60, 162.65, 159.67, 149.81, 142.53, 141.61, 133.15, 129.76, 128.66, 127.62, 128.01, 125.99, 124.68, 117.20, 116.93, 113.34, 110.73, 55.89, 36.88. Elemental analysis of $C_{26}H_{21}NO_4S_2$: calcd. C 60.64, H 4.65, N 3.07, S 14.08; found: C 60.67, H 4.44, N 2.97, S 13.42.

2-(4-Hydroxystyryl)-3-methylbenzothiazol-3-ium naphthalene-2 sulfonate (OHB-N2S): 2,3-Dimethylbenzothiazol-3-ium naphthalene-2-sulfonate (1.00 g, 2.7 mmol) and 4-hydroxybenzaldehyde (0.33 g, 2.7 mmol) were dissolved in methanol (7 mL) and piperidine (0.03 mL, 0.3 mmol). The solution was stirred at 50 °C for 6 h. The precipitated powder was filtered and purified by recrystallization in methanol. An orange powder was obtained. Yield = 34%. ^{1}H NMR (400 MHz, DMSO- d_6, δ): 10.63 (s, 1H, C_6H_5O), 8.37 (d, 1H, J = 8 Hz, C_6H_4), 8.19 (d, 1H, J = 8.4 Hz, C_6H_4), 8.14 (d, 1H, J = 15.6 Hz, C_2H_2), 8.13 (s, 1H, $C_{10}H_7SO_3^-$), 7.95 (d, 2H, J = 8.4 Hz, C_6H_5O), 7.86 (m, 2H, $C_{10}H_7SO_3^-$), 7.84 (t, 1H, J = 7.6 Hz, C_6H_4), 7.81 (d, 1H, J = 15.6 Hz, C_2H_2), 7.75 (t, 1H, J = 7.6 Hz, C_6H_4), 7.70 (d, 2H, J = 8.4 Hz, $C_{10}H_7SO_3^-$), 7.51 (m, 2H, $C_{10}H_7SO_3^-$), 6.94 (d, 2H, J = 8.8 Hz, C_6H_5O), 4.30 (s, 3H, $C_4H_3SN^+$). ^{13}C NMR (DMSO-d_6, δ): 171.76, 161.90, 149.00, 145.41, 141.72, 132.47, 132.34, 131.91, 128.95, 128.22, 127.84, 127.22, 127.07, 126.19, 126.06, 125.16, 123.80, 116.36, 116.14, 109.86, 36.05. Elemental analysis of $C_{26}H_{21}NO_4S_2$: calcd. C 65.66, H 4.45, N 2.95, S 13.48; found: C 65.65, H 4.44, N 2.97, S 13.42.

2-(4-Hydroxystyryl)-3-methylbenzothiazol-3-ium 4-chlorobenzenesulfonate (OHB-CBS): 2,3-Dimethylbenzothiazol-3-ium 4-chlorobenzenesulfonate (15.00 g, 42.2 mmol) and 4-hydroxybenzaldehyde (5.15 g, 42.2 mmol) were dissolved in methanol (200 mL) and piperidine

(0.83 ml, 8.4 mmol) was added. The solution was stirred at 50 °C for 93 h. The precipitated powder was filtered and purified by recrystallization in methanol. An orange powder was obtained. Yield = 67%. ^{1}H NMR (400 MHz, DMSO-d_6, δ): 10.63 (s, 1H, C_6H_5O), 8.38 (d, 1H, *J* = 8 Hz, C_6H_4), 8.19 (d, 1H, *J* = 8.4 Hz, C_6H_4), 8.14 (d, 1H, *J* = 16 Hz, C_2H_2), 7.95 (d, 2H, *J* = 8.8 Hz, $C_6H_4ClSO_3^-$), 7.84 (t, 1H, *J* = 8.1 Hz, C_6H_4), 7.81 (d, 1H, *J* = 15.6 Hz, C_2H_2), 7.75 (t, 1H, *J* = 7.6 Hz, C_6H_4), 7.58 (d, 2H, *J* = 8.4 Hz, $C_6H_4ClSO_3^-$), 7.37 (d, 2H, *J* = 8.8 Hz, C_6H_5O), 6.93 (d, 2H, *J* = 8.8 Hz, C_6H_5O), 4.30 (s, 3H, $C_4H_3SN^+$). ^{13}C NMR (DMSO-d_6, δ): 171.58, 161.68, 148.80, 146.83, 141.54, 132.47, 132.14, 128.76, 127.66, 127.26, 127.03, 124.96 123.68, 116.17, 115.94, 109.68, 35.85. Elemental analysis of $C_{22}H_{18}ClNO_4S_2$: calcd. C 57.45, H 3.94, N 3.05, S 13.94; found: C 50.21, H 3.47, N 2.86, S 12.75.

2-(4-Hydroxystyryl)-3-methylbenzothiazol-3-ium 4-methylbenzenesulfonate (OHB-T): 2,3-Dimethylbenzothiazol-3-ium 4-methylbenzenesulfonate (5.00 g, 14.9 mmol) and 4-hydroxybenzaldehyde (1.82 g, 14.9 mmol) were dissolved in methanol (60 mL) and piperidine (0.15 mL, 1.5 mmol). The solution was stirred at 50 °C for 24 h. The precipitated powder was filtered and purified by recrystallization in methanol. A yellow powder was obtained. Yield = 53%. ^{1}H NMR (400 MHz, DMSO-d_6, δ): 10.61 (s, 1H, C_6H_5O), 8.38 (d, 1H, *J* = 8 Hz, C_6H_4), 8.19 (d, 1H, *J* = 8.4 Hz, C_6H_4), 8.14 (d, 1H, *J* = 15.6 Hz, C_2H_2), 7.95 (d, 2H, *J* = 8.8 Hz, $C_7H_7SO_3^-$), 7.84 (t, 1H, *J* = 7.3 Hz, C_6H_4), 7.81 (d, 1H, *J* = 15.6 Hz, C_2H_2), 7.75 (t, 1H, *J* = 7.6 Hz, C_6H_4), 7.47 (d, 2H, *J* = 8 Hz, $C_7H_7SO_3^-$), 7.10 (d, 2H, *J* = 8.4 Hz, C_6H_5O), 6.94 (d, 2H, *J* = 8.8 Hz, C_6H_5O), 4.30 (s, 3H, $C_4H_3SN^+$), 2.28 (s, 3H, $C_7H_7SO_3^-$). ^{13}C NMR (DMSO-d_6, δ): 171.82, 161.85, 149.02, 145.51, 141.75, 137.37, 132.36, 128.98, 127.85, 127.22, 125.29, 125.21, 123.89, 116.41, 116.14, 109.95, 36.08, 20.85. Elemental analysis of $C_{23}H_{21}NO_4S_2$: calcd. C 62.85, H 4.82, N 3.19, S 14.59; found: C 62.85, H 4.82, N 3.14, S 14.62.

B. Quantitative Powder SHG Test

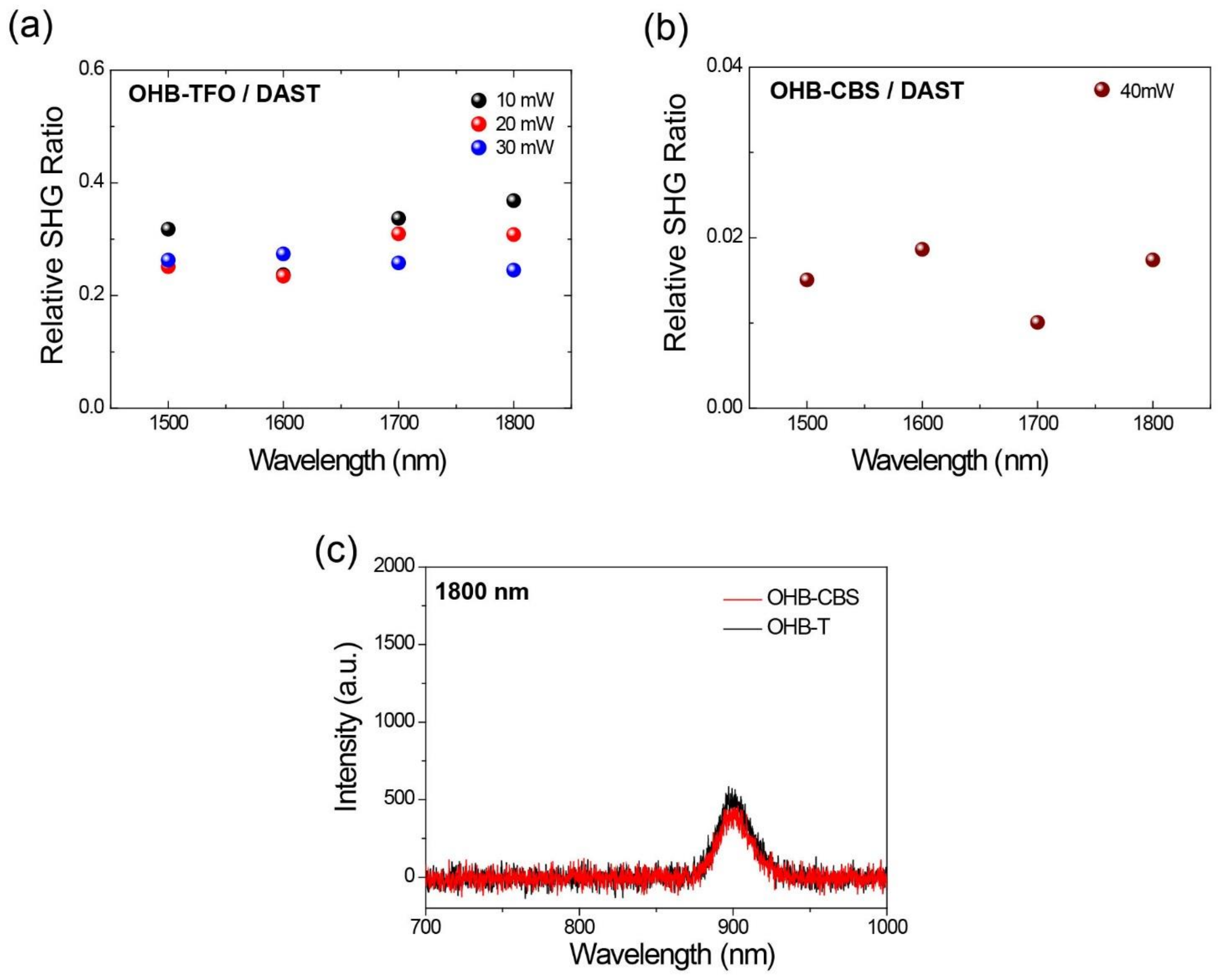

Figure S1. Quantitative powder SHG test at a fundamental pump wavelength of 1500–1800 nm. Relative SHG intensity of (a) OHB-TFO and (b) OHB-CBS powders relative to DAST powder. (c) Spectra of OHB-CBS and OHB-T powders obtained with 1800-nm pumping.

C. Crystal Structures

OHB-TFO: OHB-TFO single crystals were grown from methanol solution by slow evaporation. $C_{23}H_{18}F_3NO_5S_2$, M_r = 509.5, monoclinic, space group *Cc*, *a* = 7.9977(6) Å, *b* = 21.2304(17) Å, *c* = 13.8142(13) Å, β = 106.815(2)°, *V* = 2245.3(3) Å^3, *Z* = 4, *T* = 290(1) K, μ(MoKα) = 0.299 mm^{-1}. Of the 10824 reflections collected in the θ range of 3.08–27.45°, using ω scans on a Rigaku R-axis Rapid S diffractometer, 4694 were unique reflections (R_{int} = 0.0382). The structure was solved and refined against F^2 using SHELXL-2014/7 [G. M. Sheldrick, Acta Cryst. C71, 3 (2015)] with 308 variables, wR_2 = 0.1652, R_1 = 0.0467 ($Fo^2 > 2\sigma(Fo^2)$), GOF = 1.063, and max/min residual electron density 0.510/–0.353 eÅ^{-3} (CCDC-1885049).

OHB-MBS: OHB-MBS single crystals were grown by a rapid cooling method in methanol solution. $C_{16}H_{14}NOS\cdot C_7H_7O_4S$, M_r = 455.53, monoclinic, space group *C*2/*c*, *a* = 30.0749(16) Å, *b* = 10.8859(5) Å, *c* = 12.8933(6) Å, β = 90.335(1)°, *V* = 4221.1(4) Å^3, *Z* = 8, *T* = 150(1) K, μ(MoKα) = 0.289 mm^{-1}. Of the 20095 reflections collected in the θ range 3.2–27.5° using ω scans on a Rigaku R-axis Rapid S diffractometer, 4821 were unique reflections (R_{int} = 0.0189). The structure was solved and refined against F^2 using SHELXL-2017/1 [G. M. Sheldrick, Acta Cryst. C71, 3 (2015)], 283 variables, wR_2 = 0.0893, R_1 = 0.0333 ($Fo^2 > 2\sigma(Fo^2)$), GOF = 1.046, and max/min residual electron density 0.465/–0.392 eÅ^{-3} (CCDC-1991124).

OHB-CBS: OHB-CBS single crystals were grown by the slow cooling method in methanol solution. $C_{22}H_{18}ClNO_4S_2$, M_r = 459.94, orthorhombic, space group *Pca*2$_1$, *a* = 15.6229(4) Å, *b* = 7.0324(2) Å, *c* = 19.1407(4) Å, *V* = 2102.92(9) Å^3, *Z* = 4, *T* = 290(1) K, μ(MoKα) = 0.410 mm^{-1}. Of the 19296 reflections collected in the θ range 3.2–27.5° using ω scans on a Rigaku R-axis Rapid S diffractometer, 4589 were unique reflections (R_{int} = 0.0225). The structure was solved and refined against F^2 using SHELXL-2017/1 [G. M. Sheldrick, Acta Cryst. C71, 3 (2015)], with 274 variables, wR_2 = 0.0847, R_1 = 0.0304 ($Fo^2 > 2\sigma(Fo^2)$), GOF = 1.100, and max/min residual electron density 0.240/–0.179 eÅ^{-3} (CCDC-1827729).

D. Macroscopic Optical Nonlinearity

The microscopic and macroscopic optical nonlinearity of OHB-TFO was estimated from the quantitative SHG powder test measurements in the following way. The direction of the maximal first hyperpolarizability ($\beta_{\max}$) of OHB chromophores, i.e. the direction of the main charge-transfer axis, is assumed to be between the O atom on the electron donor and the C atom between the S and N atoms on the electron acceptor (see the red arrows in Figure 2 in the manuscript). From the X-ray crystal structure, the direction of $\beta_{\max}$ makes an angle of $\theta_p = 10.0°$ (ordering angle) with respect to the mirror symmetry plane. Considering the so-called 1D chromophore approximation [*Phys. Rev.* **1982**, *A26*, 2028], the diagonal component of the effective hyperpolarizability tensor of the OHB-TFO crystals (along the polar axis of the crystal in the *ac* crystallographic plane) can be estimated as $\beta_{333}^{\mathrm{eff}} = \beta_{\max} \cos^3 \theta_p$ and the off-diagonal component as $\beta_{223}^{\mathrm{eff}} = \beta_{\max} \cos \theta_p \sin^2 \theta_p$.

The value of the maximal first hyperpolarizability ($\beta_{\max}$) is estimated from the quantitative powder SHG measurements in the range of 1500–1800 nm (Figure S1), which results in the experimental ratio of the non-resonant SHG efficiencies $\eta_{\mathrm{OHB\text{-}TFO}}/\eta_{\mathrm{DAST}} = 0.28 \pm 0.04$. The SHG powder test efficiency η depends on the effective hyperpolarizability tensor ($\beta_{ijk}^{\mathrm{eff}}$) of each crystal and the number density of chromophores N and is proportional to $N^2 \langle (\beta^{\mathrm{eff}})^2 \rangle$, where $\langle (\beta^{\mathrm{eff}})^2 \rangle$ is the squared effective hyperpolarizability averaged considering the crystal symmetry according to Reference [*J. Appl. Phys.* **1968**, *39*, 3798]; see also Reference [*CrystEngComm*, **2016**, *18*, 7180]. For DAST, $N^2 \langle (\beta^{\mathrm{eff}})^2 \rangle$ is evaluated considering the hyperpolarizability tensor components as determined by quantum chemical calculations in Reference 27 [*CrystEng-Comm* **2011**, *13*, 444]. From the experimentally determined ratio of SHG efficiencies we are able to calculate the corresponding value of $\beta_{\max}$ for the OHB chromophore, resulting in $\beta_{\max} = (100 \pm 7) \times 10^{-30}$esu. The diagonal component of the effective hyperpolarizability tensor of the OHB-TFO crystals is then about $\beta_{333}^{\mathrm{eff}} = \beta_{\max} \cos^3 \theta_p$ ~ 96 $\times 10^{-30}$ esu, whereas the largest off-diagonal component is about $\beta_{223}^{\mathrm{eff}} = \beta_{\max} \cos \theta_p \sin^2 \theta_p$ ~ 3 $\times 10^{-30}$ esu.
